# Increasing physical activity in office workers – the Inphact Treadmill study; a study protocol for a 13-month randomized controlled trial of treadmill workstations

**DOI:** 10.1186/s12889-015-2017-6

**Published:** 2015-07-10

**Authors:** Frida Bergman, Carl-Johan Boraxbekk, Patrik Wennberg, Ann Sörlin, Tommy Olsson

**Affiliations:** Department of Public Health and Clinical Medicine, Medicine, Umeå University, Umeå, Sweden; CEDAR, Center for Demographic and Aging Research, Umeå University, Umeå, Sweden; UFBI, Umeå Center for Functional Brain Imaging, Umeå University, Umeå, Sweden; Department of Public Health and Clinical Medicine, Family Medicine, Umeå University, Umeå, Sweden; Department of Community Medicine and Rehabilitation, Physiotherapy, Umeå University, Umeå, Sweden

**Keywords:** Sedentary behaviour, Physical activity, Non-Exercise Activity Thermogenesis, Treadmill workstation, Randomized controlled trial, Workplace, Obesity prevention

## Abstract

**Background:**

Sedentary behaviour is an independent risk factor for mortality and morbidity, especially for type 2 diabetes. Since office work is related to long periods that are largely sedentary, it is of major importance to find ways for office workers to engage in light intensity physical activity (LPA). The Inphact Treadmill study aims to investigate the effects of installing treadmill workstations in offices compared to conventional workstations.

**Methods/Design:**

A two-arm, 13-month, randomized controlled trial (RCT) will be conducted. Healthy overweight and obese office workers (n = 80) with mainly sedentary tasks will be recruited from office workplaces in Umeå, Sweden. The intervention group will receive a health consultation and a treadmill desk, which they will use for at least one hour per day for 13 months. The control group will receive the same health consultation, but continue to work at their regular workstations. Physical activity and sedentary time during workdays and non-workdays as well as during working and non-working hours on workdays will be measured objectively using accelerometers (Actigraph and activPAL) at baseline and after 2, 6, 10, and 13 months of follow-up. Food intake will be recorded and metabolic and anthropometric variables, body composition, stress, pain, depression, anxiety, cognitive function, and functional magnetic resonance imaging will be measured at 3–5 time points during the study period. Interviews with participants from the intervention group will be performed at the end of the study.

**Discussion:**

This will be the first long-term RCT on the effects of treadmill workstations on objectively measured physical activity and sedentary time as well as other body functions and structures/morphology during working and non-working hours among office workers. This will provide further insight on the effects of active workstations on our health and could fill in some of the knowledge gaps regarding how we can reduce sedentary time in office environments.

**Trial registration:**

ClinicalTrials.gov Identifier NCT01997970, 2nd Nov 2013.

## Background

In addition to physical inactivity, sedentary behaviour has emerged as an independent risk factor for morbidity and mortality [1]. Sedentary behaviour is defined as activities with an energy expenditure of ≤1.5 metabolic equivalents while in a sitting or reclining posture [2]. A recent meta-analysis showed that a high amount of sedentary time significantly increases the risk of type 2 diabetes, all-cause mortality, and cardiovascular disease incidence and mortality as well as cancer incidence and mortality [3].

Non-exercise activity thermogenesis (NEAT) is the energy expended during non-exercise activities, i.e. the energy that we expend during light intensity physical activities (LPA). These activities are often performed at low intensities during our everyday lives and include walking, climbing stairs, fidgeting, or cleaning the house. This is separate from the energy expended during moderate-to vigorous physical activity (MVPA) like aerobic or resistance exercises [4]. Notably, the health risks associated with sedentary behaviour are independent of the amount of MVPA [3], demonstrating the relevance of interventions that reduce sedentary time and increase LPA, aside from interventions trying to increase the amount of MVPA.

Due to its low intensity, it is possible to perform LPA during large parts of the day. Thus, in contrast to MVPA, which involves a higher intensity level and often is restricted to a limited number of hours per day, LPA could contribute a larger extent to the total daily energy expenditure [5].

Occupation plays a major role in the daily amount of NEAT. The prevalence of jobs based on sedentary tasks in the US has continuously increased since the 1960’s, while more physically demanding jobs have decreased in prevalence [6]. Studies from Australia, Scotland, and Sweden have shown that office workers spend between 66-82 % of their work hours sedentary [7–10]. Furthermore, sedentary time seems to be higher on workdays compared to non-workdays [8, 10, 11] as well as during working hours compared to non-working hours on workdays [10]. Church et al. concluded that the drop in energy expenditure due to an occupational change from active to sedentary jobs is a main reason for the increase in overweight and obesity over the past decades [6]. Workplaces are important targets when it comes to reducing sedentary behaviour [6–8]. If we could find ways to decrease sedentary time in the workplace and replace it with LPA, increasing NEAT, health gains are likely to be gained.

A key element to make the environment in offices more activity-permissive may be the implementation of active workstations. This consists of treadmills or other devices, such as bicycles or stepping devices that the employees use while working as usual at their desk. A recent review concluded that treadmill workstations can have positive effects on sitting time, energy expenditure, and different health parameters such as hip and waist circumference, weight, body fat percentage, high density lipoprotein (HDL), low density lipoprotein (LDL), and total cholesterol [12]. However, earlier studies of treadmill workstations were either of rather short duration (up to 12 weeks) [13, 14], lacked a control-group [15, 16], or included a control group that was followed for a shorter time period than the intervention group [17]. Importantly, no randomized controlled trials (RCTs) have investigated the long-term impact of treadmill workstations on reducing sitting time during working hours [18].

### Objectives

The Inphact Treadmill study is a 13-month RCT aimed at studying the long-term effects of installing active workstations in office environments. Subjects included will be overweight or obese, as subjects with overweight or obesity have higher health risks associated with prolonged sitting time compared to normal weight subjects [19].

### Theoretical basis

Different factors based on individual, environmental, social, and organizational levels influence our tendency and ability to be active [5, 20]. Attributes in the environment, such as the walkability of the neighbourhood or the design of workplace furniture, act together with individual preferences and social norms in determining sedentary behaviour in different settings [20]. Owing to changes that have occurred in our environment over the past decades, we have “been seduced” into becoming more sedentary in our everyday lives. This increase in sedentary behaviour is having effects on our health. If people are to become more active, the environment needs to be permissive. We need to work with environmental factors and investigate how we can reduce sitting time in different settings [5] in addition to working with individual and social factors. One such important setting is the workplace.

The Inphact Treadmill-study is a complex intervention, working with factors based on individual, social, and environmental levels in the workplace. We expect that changing the (individual’s) environment by installing treadmill workstations in offices, people will sit less and become more active during their everyday life.

### Hypotheses

The primary hypothesis is that the intervention group will increase their time spent walking compared to the control group. The secondary hypotheses are that the intervention group also will increase their time spent standing, decrease their time spent sitting, and increase their daily LPA compared to the control group. No change in MVPA is expected.

**Fig. 1:**
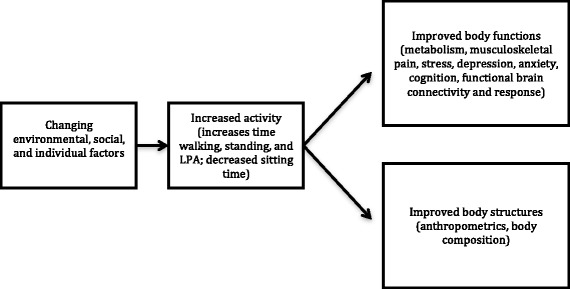
Theoretical model of the study. Our primary hypothesis is that by working with factors based on environmental, individual, and social levels, walking time will increase in the intervention group compared to the control group. The secondary hypotheses are that time spent sitting will decrease and time spent standing, as well as daily light physical activity (LPA), will increase. These changes in activity will in turn have effects on different body functions and body structures. No change in moderate-to vigorous physical activity is expected

This increase in activity and decrease in sedentary time will lead to positive effects on body functions including metabolism, musculoskeletal pain, stress, depression, anxiety, cognition, and functional brain connectivity and response, as well as have an effect on body structures including anthropometrics and body composition (Fig. 1).

## Methods

### Trial design

The Inphact Treadmill study is a two-arm, randomized, controlled study. Participants will be individually randomized into either an intervention group or a control group with an allocation ratio of 1:1. The study will be conducted and reported in accordance with the CONSORT guidelines.

The Inphact Treadmill study is funded by Umeå University.

Ethical approval was granted by the Regional Ethical Review Board, Umeå, Sweden.

### Trial status

Recruitment and screening of participants has been completed at the time of manuscript submission. Data collection has started, and is ongoing at this time.

### Participants

#### Settings and locations

The study will take place in Umeå, Northern Sweden. Umeå is located at latitude N 63° and has major seasonal variations, with cold winters and relatively warm summers. Forty of the participants (20 from each group) will start the study in April 2014 and 40 participants (20 from each group) will start the study in October 2014. Potential seasonal variations in outcome variables can thus be estimated.

Management personnel at the different office workplaces will first be contacted with information about the study and a presentation on the risks of sedentary behaviour. If granted by management, the research team will visit the companies to give an informational presentation about the study to the employees. At this visit, the employees will be thoroughly informed about the study and about the commitment expected of those participating. Those employees who can not attend this information session, but who shows interest in participating in the study, will receive the same information via telephone.

Employees from private companies as well as those from state, county, and municipal offices will be included in the study group. The settings in the different companies range from private offices to open office settings.

#### Eligibility criteria

Participants aged between 40–67 years, with a body mass index (BMI) between 25–40 kg/m^2^, and having an office job consisting of mainly sedentary tasks are eligible. Exclusion criteria includes diabetes mellitus, exhaustion disorder, moderate or severe depression or anxiety, severe kidney disease, severe cardiovascular disease, severe gastrointestinal or lung disease, untreated thyroid disease, pregnancy, a previous cardiovascular event such as transient ischemic attack/stroke, more than 6 % weight loss during the previous 6 months, engagement in extensive aerobic exercise training, or musculoskeletal pain making it difficult to walk on a treadmill. Participants are also excluded if they have more than one day of travel per work week or have plans to leave the organization during the study period.

For the functional magnetic resonance imaging (fMRI) measurements, exclusion criteria includes implanted metal or electrical devices, extraneous metal pieces in the body, metal splinters, claustrophobia, neurological diseases, or tattoos containing lead.

#### Screening process

Employees interested in participating in the study will initially be screened for the inclusion and exclusion criteria via a telephone interview performed by a member of the research team. Those employees who are eligible and willing to participate in the study will then be called to the University hospital in Umeå to provide signed consent and to go through a more thorough screening process. At this visit, a clinical examination will be performed by a doctor at the clinical research centre, and routine blood samples will be taken for haemoglobin and plasma glucose/HbA1c levels and kidney and liver function. An experienced nurse will measure pulse rate, blood pressure, weight, and height.

At the screening visit, the participants will also complete questionnaires including the Hospital Anxiety and Depression scale (HAD scale), self-rated Exhaustion Disorder (s-ED), and the Workforce Sitting Questionnaire (WSQ) with regards to the exclusion criteria. The HAD scale is a commonly used clinically questionnaire and has been found to be valid for assessing symptom severity and caseness of depression and anxiety [21]. Based on clinical expertise, moderate-to-severe depression is suspected when scoring more than 10 points on the depression part of the scale, and moderate to severe anxiety is suspected when scoring more than 10 points on the anxiety part of the scale. The s-ED scale (the Institute for Stress Medicine, Gothenburg) is a four-item scale that follows a predefined scoring protocol. This questionnaire has good construct and predictive validity [22]. In the WSQ, as described by Chau et al. [23], the participants estimate how much time they spent sitting in different domains during workdays and non-workdays during the past 7 days. This questionnaire has acceptable reliability and validity [23].

### Interventions

#### Health consultation

The participants in both groups will receive an individual health consultation in the beginning of the study, which will be led by an experienced nurse. This consultation will be individually adapted and the participants will receive feedback on some of the measurements taken at screening and at baseline. The feedback includes weight circumference, BMI, blood pressure, and cholesterol and HbA1c-levels. Also included is feedback about their amount of sedentary time, which was subjectively estimated based on the WSQ at screening. This will be followed by information and discussion about physical activity and diet according to the guidelines used in the Västerbotten County Council (VLL). The participants’ own reflections and thoughts about their baseline values and their current life situation will then be discussed with the nurse. The health consultation will take about 30–40 min.

Written information folders about healthy lifestyles will be distributed to the participants. The information folder about physical activity emphasises the need for at least 30 min of moderate physical activity per day. To improve aerobic capacity and strength, the folder recommends exercise at a vigorous level 20–60 min two to three times per week. A few examples of how the participants can improve their everyday LPA to increase NEAT are also given in this folder, e.g. to take the stairs instead of the elevator, to walk or take a bike to and from work, to stand up while talking on the phone, or to try “walk and talk-meetings”.

Dietary recommendations will be provided in separate folders. An information folder is directed towards recommendations about diet for subjects with high blood lipid levels. An additional dietary health guide gives further recommendations about diet, especially for saturated, unsaturated and polyunsaturated fat, cholesterol, and trans fats, with suggested recipes. The information also includes other lifestyle factors, such as alcohol and smoking.

#### Treadmill workstation

Participants in the intervention group will receive, apart from the health consultation, a treadmill workstation consisting of a treadmill adapted for desk use, which they will use for 13 months. This equipment will be installed in place of the subject’s regular working desk with a height-adjustable table. A physiotherapist will perform the instalment of the treadmill workstations and give ergonomic advice. Participants will be instructed on how to use the treadmill at a self-chosen speed and to gradually increase their time using the treadmill, starting with 15 min twice daily during the first week. After the initialization process, they will be instructed to use the treadmill for at least one hour per day, but preferably longer. Whenever they want to sit down during the day, the treadmill can be tipped sideways so that the desk can be lowered to chair level.

Participants in the intervention group receive booster e-mails from the research group at 4 time points (after 5–6 weeks, 19–20 weeks, 31 weeks, and 50 weeks) during the study period. In these booster e-mails, subjects receive information about the negative health effects of sitting, are inspired to use the treadmill as much as they can, and are reinforced to meet the criteria of at least one-hour of walking time per day. Participants in the control group will not receive any booster emails during the study period.

### Outcomes

**Fig. 2:**
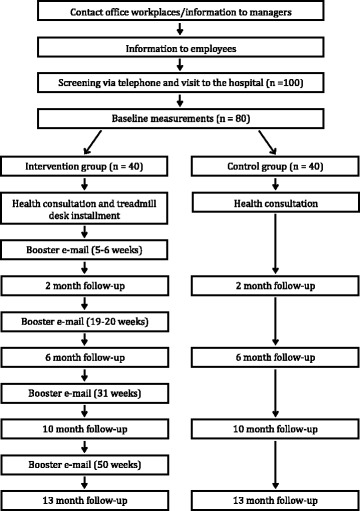
Overview of the study plan. Measurements taken at each follow-up can be seen in Table 1

The study duration is 13 months. Outcomes will be assessed at three to five time points: at baseline, 2 months, 6 months, 10 months, and 13 months. Figure 2 and Table 1 show the study design and the assessment points, respectively, for the different outcome measurements.

**Table 1:** Measurements at baseline and each follow-up for both groups

Measurement	Baseline	2 months	6 months	10 months	13 months
Physical activity, time spent walking, standing, and sitting	x	x	x	x	x
Diet	x	x	x	x	x
Musculoskeletal pain	x	x	x	x	x
Anthropometry	x		x		x
Body composition	x		x		x
Metabolism	x		x		x
Stress	x		x		x
Depression, anxiety	x		x		x
Cognitive functions	x		x		x
fMRI	x				x
Interview					x

#### Demographic data

At baseline, demographic data including age, gender, marital status, educational attainment, and number of children will be asked for. Any new health problems, added medications and/or altered doses of already existing medications during the past 3 months will also be recorded at baseline and at 2, 6, 10, and 13 months. At the follow-ups, participants will also be asked to report any life events that may have occurred during the last 3 months that might possibly affect their health or wellbeing.

#### Self-rated health

At baseline and at the 2-, 6-, 10-, and 13-month follow up visits, self-rated health will be obtained by two questions from the Swedish version of the RAND-36 (Lotti Orwelius et al., unpublished observations); “In general, would you say your health is…” and “Compared to one year ago, how would you rate your health in general now?”.

#### Sedentary time and physical activity

Daily light, moderate, and vigorous physical activity will be measured objectively with the tri-axial Actigraph wGT3x-BT activity monitor (ActiGraph, Pensacola, Florida, USA) at baseline and at the 2-, 6-, 10-, and 13-month follow-up visits. This monitor will be worn around the waist above the right hip with an elastic belt. Participants are asked to use the Actigraph during all their waking hours, except during water-based activities, for 14 consecutive days during each assessment period. The raw data will be collected at 30Hz.

Sitting/lying (reported as sitting), standing, and walking time, number of sedentary bouts, number of breaks from a sedentary bout, and total number of steps and cadence will be measured objectively with the activPAL3 and activPAL3 micro activity monitor (PAL Technologies Limited, Glasgow, UK; default settings) at baseline and at the 2-, 6-, 10-, and 13-month follow-up visits. The participants will wear one of these devices fastened to the anterior mid-line of the right thigh using Mepore surgical dressing. activPAL has been proven to provide valid, reliable, and sensitive measurements on changes in body postures and motion [24–27], and in estimating breaks from sedentary time [27]. Participants will be asked to use the activPAL for 24 h, except during water-based activities, for the first seven days of each assessment period when they are simultaneously wearing the Actigraph.

During each measurement period, participants will report the time that they usually go to sleep and wake up on workdays and non-workdays, as well as the usual time that they arrive at and leave work. This will be used to calculate the variables from the activity devices during the entire day on both work and non-work days, as well as dividing the activity into working hours and non-working hours during work days. Participants will also be asked to report any period of not wearing the monitors.

Self-reported physical exercise will be recorded at baseline and at the 2-, 6-, 10-, and 13-month follow-up visits with the question, “how many days during the past 3 months have you exercised in workout clothes, with the purpose of improving your fitness and/or to feel good?” They will also be asked to report if, and if so how, they have made any changes in their daily activity habits during work, leisure time, and/or transportation during the past 3 months.

#### Diet assessment

Participants will be asked to register their dietary intake using a food diary at baseline and at the 2-, 6-, 10-, and 13-month follow-up visits. This will be done on four of the days while they are wearing the Actigraph; three weekdays and one day during the weekend. The participants will be instructed by an experienced dietician to register their entire energy intake, specifying the type and amount of food and drink that they consume during these four days, estimated using a portion guide.

#### Anthropometric outcomes

Anthropometric measurements will be taken at baseline and at the 6- and 13-month follow-up visits. This will be done at the clinical research centre at the University hospital in Umeå, Sweden, using standardized measurements. During these measurements, the participants will wear light indoor clothing without shoes.

Body height will be measured to the nearest 0.1 cm using a wireless stadiometer (Seca 264; Seca ltd, Hamburg, Germany). Body weight will be measured to the nearest 0.1 kg using a calibrated electronic digital scale (Tanita BWB-800 MA; Umedico AB, Rosersberg, Sweden). BMI is calculated as body weight in kilograms divided by body height in meters squared (kg/m^2^).

Waist and hip circumference will be measured with a tape measure to the nearest 0.5 cm. Waist circumference will be measured midway between the lower rib margin and the iliac crest during a light exhale. Hip circumference will be measured over the widest parts of the hips.

Sagittal abdominal diameter will be measured (Oscar Instrument AB, Hyssna M, art.nr. 7–506, Kungälv, Sweden) at the umbilical level, with the participants lying on their backs with extended legs.

Blood pressure will be measured twice at each follow-up visit on the right arm with the participant in a seated position. Pulse will be measured manually at the right wrist twice. The mean value of the blood pressure and pulse measurements will be reported.

#### Body composition

Body composition will be measured with Dual X-ray absorptiometry (DXA) (Lunar Prodigy X-ray Tube Housing Assembly, Brand BX-1 L, Model 8743; GE Medical Systems, Madison, WI, USA) by an experienced nurse at baseline and at the 6- and 13-month follow-up visits at the clinical research centre. Absolute values of fat mass and lean mass, as well as android and gynoid fat mass, will be measured for each participant.

#### Metabolic outcomes

Basal blood samples will be drawn after an overnight fast by an experienced nurse at baseline and at the 6- and 13-month follow-up visits at the clinical research centre. Participants will be instructed to avoid high-intensity exercise and stressful situations on the day before the tests, and to avoid nicotine and caffeine on the morning before the test. Plasma cholesterol, triglycerides, HDL, and LDL and total cholesterol, HbA1c-level, high sensitivity C-reactive protein, and liver enzymes will be measured in the blood samples.

##### Insulin sensitivity/glucose levels

For measurements of insulin sensitivity/glucose levels, an oral glucose tolerance test will be performed after an overnight fast. Plasma insulin and glucose levels will be measured at baseline then at 30-min intervals for up to 120 min after oral intake of 75 g of glucose.

#### Musculoskeletal pain

Musculoskeletal pain from the head, neck, shoulders, thoracic spine, lumbar back, hips, knees, and feet during the past 3 months will be measured at baseline and at the 2-, 6-, 10-, and 13-month follow-up visits using the Chronic Pain Grade Questionnaire (CPGS) [28]. Participants will complete the questionnaire at home. The CPGS has been shown to be a valid questionnaire [28, 29], and includes subscales for both pain severity and pain-related disability. The subscale scores are combined into a grade, classifying the participants into 5 categories ranging between 0 (no pain problem) to 4 (severe interference). An additional question was recently added to the questionnaire, which asks for the number of days of pain during the past 6 months. Pain is also classified as non-persistent (1–89 pain days) or persistent (90–180 pain-days) [30].

#### Stress

Saliva samples for cortisol measurements and stress scales will be performed at baseline and at the 6- and 13-month follow-up visits.

Saliva samples will be taken at 7 am, 11 am, 4 pm, and 11 pm during one workday. The participants are instructed to avoid high-intensity exercise on the day before as well as during the test day. They are also instructed not to eat, use nicotine, or brush their teeth one hour before the test. Premenopausal women are instructed to take the samples on the seventh day after the start of their menstruation. The participants will take the samples at home using Salivette® tubes. They are instructed to chew on the included swab for one minute or to keep the swab under their tongue for one and a half minutes. The swab is then put back into the tube and the lid is put on for storage. They will be instructed to keep the saliva samples in the refrigerator for up to one day before handing them over to the research team. The saliva samples will be analysed at the department of Clinical Chemistry, Umeå University hospital.

Self-reported stress during the past week will be measured using the Stress- and Energy scale (SE scale) [31]. The participants will complete the questionnaire at home. This scale consists of 12 adjectives of which 6 cover the dimensions of stress and 6 cover the dimension of energy. Answers are given on a 6-point scale (0–5) ranging from “not at all” to “extremely”.

#### Depression, anxiety

The participants’ feelings of depression and anxiety during the past week will be measured at baseline and at the 6- and 13-month follow-up visits using the HAD scale [21]. The participants will fill out the questionnaire at home.

#### Cognitive functions

A cognitive test battery covering functions including episodic memory, working memory, and executive functions will be administered at 0, 6, and 13 months. The tests will be performed at Umeå University during office hours. The total time for the cognitive tests will be approximately 45 min. The selected tests are a mixture of computerized tests and pen and paper tests. Below are descriptions of each test and the order of the tests.

##### Free recall, episodic memory [32]

In free recall, the participants will be presented with a list of 16 words on a computer screen at the rate of three seconds per item. When the list is completed, a free recall test will follow (maximum 2 min) where the participants will be asked to recall as many of the previously presented words as possible. The dependent variable will be the number of correctly recalled words.

##### N-back, executive function [33]

In n-back, the participants will be asked to indicate whether each number (1–9) in a list presented on a computer matches a number occurring one, two, or three numbers back. Accuracy and response time will be used as dependent measures.

##### Encoding, episodic memory (recognition)

In this test, the participants will be presented with a list of 30 words on a computer one at a time for three seconds per item. The task is to encode each word for a subsequent memory test (retrieval-recognition; see below).

##### Flanker test, executive function [34]

In the Eriksen flanker task, the participants will indicate the direction of the middle arrow in either congruent (>>>>>) or incongruent (> > <>>) stimuli presented on a computer by pressing a button as fast and as accurately as possible. Accuracy and response time will be used as dependent measures.

##### Backward digit span, working memory [35]

In Digit span, the participants will be asked to recall digits backwards. The dependent variable will be the longest correctly completed number sequence.

##### Trail making A + B, working memory and executive function [36]

In the trail making test (TMT), the participants will be shown circles containing letters and numbers. They will be asked to connect the circles containing numbers in numerical order (TMT part 2) and to alternate between numbers and letters according to numerical and alphabetical order (TMT part 4). This is a pen and paper test. A shifting cost will be calculated, i.e. the difference in time (seconds) taken to complete TMT part 4 as compared to TMT part 2.

##### Retrieval-recognition, episodic memory

In this test, a new list of words will be presented to the participants. The list contains the words from the encoding phase (see above) and the task is to indicate if the words have previously been presented or not. The dependent variable will be the number of correctly recognized items.

##### Digit symbol, processing speed [35]

In digit symbol, the participants will be required to draw symbols in empty boxes, associating them with numbers according to a coding key. The number of items completed in 90 s will be used as the outcome measure.

Brain-derived neurotrophic factor (BDNF) will be measured in plasma samples from baseline and at the 6- and 13-month follow-up visits. BDNF has previously been shown to correlate to neuronal plasticity, including effects on the hippocampal volume [37].

#### Functional Magnetic Resonance Imaging (fMRI)

Twenty subjects from each study group will be randomly selected for fMRI scanning at baseline and at the 13-month follow-up. This will be performed at the Centre for Functional Brain Imaging at the University hospital in Umeå, Sweden. The participants will be instructed to eat as usual before the measurement, but to refrain from nicotine and caffeine one hour before the test and from high-intensity exercise and alcohol for twenty-four hours before the test.

High-resolution T1 images will be acquired in order to examine potential effects on grey matter volume. Furthermore, blood oxygen level dependent (BOLD) T2* images will be acquired during a 10 min rest period in order to evaluate functional resting state networks, a measure of global brain function across various ages [38]. The last task during scanning will be the Eriksen flanker task to evaluate task-specific effects on functional brain response. In the Eriksen flanker task, participants will indicate the direction of the middle arrow in either congruent (>>>>>) or incongruent (> > <>>) stimuli by pressing MRI-adapted buttons as fast and as accurately as possible. Accuracy and response time will be used as dependent measures.

#### Interview

At short-term follow-up visits, studies testing the effects of treadmill workstations have found that participants are generally positive and enthusiastic about the treadmill and would use it if available [13, 39]. A recent study investigated facilitators and barriers regarding usability, safety, comfort, and productivity in five female office workers who used treadmill workstations for 6 months [40]. By conducting an interview study after the study period is over, i.e. after 13 months, we will investigate the participants’ experiences with and attitudes towards working on a treadmill workstation, and further assess barriers and facilitators. The interview results may help with the development and implementation of active workstations and in the construction of activity-enhancing workplaces in the future.

This qualitative interview study includes individual semi-structured interviews. Approximately 15 interviews are estimated to be needed. Participants from the intervention group, who started in April 2014, will firstly be asked to participate. If more interviews are needed after this, participants from the intervention group starting in October 2014 will also be invited to participate. The interviews will be performed either at the companies where the participant works or at the university, depending on what works best for the participant. The study will be conducted using grounded theory methodology with an emergent design. The data will be processed through open, axial and selective coding, with constant comparison of the emerging codes. Using this method, our aim is to develop a theoretical model based on the gathered data. The credibility of the results will be determined by triangulation, where three members of the research team will perform the analysis independently.

### Sample size

Sample size calculation was based on two previous long-term studies on treadmill workstations available at the time the study was planned. These long-term studies found a significantly increased time spent walking per day [15, 16] after installing treadmill workstations. Based on these studies and stratification according to BMI (25–30 kg/m^2^ and 30–40 kg/m^2^), a study with 30 participants per group would have 85 % power in finding a statistically significant difference in 30 min of walking per day (SD 60, 8 min) between the groups. To compensate for an estimated dropout rate of 30 %, 39 individuals per group will be needed. Thus, 80 participants in total will be recruited for the Inphact Treadmill-study.

### Randomization

After the baseline measurements, participants will be individually randomized by an external statistician into one of the two groups, with 40 participants per group. They will be stratified according to BMI (25–30 kg/m^2^ and 30–40 kg/m^2^).

For the fMRI-measurement, 20 of the eligible participants from each group will be randomly selected to participate.

### Blinding

Researchers responsible for gathering, compiling, and assessing outcomes will be blinded with regards to the group allocations.

### Statistical methods

Primary analyses will follow intention-to-treat principles. Changes over time between the two study arms and across the different data collection time points will be analysed using methods that account for within-subject dependencies, e.g. mixed models. The threshold for statistical significance will be set at *p* <0.05. Adjustments will be made for baseline characteristics and potential confounding variables, such as age and gender.

## Discussion

Sedentary time has increased in a major proportion of the population, with associated negative health effects [3]. Finding methods that can reduce sedentary time on a long-term basis in settings where sedentary time is often high, such as offices, can have major effects in improving health on a population level. The Inphact Treadmill study could help fill in some of the existing gaps in our knowledge about the long-term effects that the instalment of active workstations could have on behaviour and different health parameters. This will be done through the extensive measurements of activity levels, body functions, and body structures. By using qualitative interviews, the study could also provide further insight and guidance on how the implementation process could most preferably be handled in future research and among companies wanting to implement these active workstations.

A major strength of our study is the use of objective measurements of sedentary behaviour and physical activity. By using both an inclinometer and an accelerometer, we will obtain a valid measurement with detailed information about these different behaviours, something that we would not be able to capture using only one of these devices alone. These two devices complement each other and provide a comprehensive picture of how active and sedentary is a person. We also investigate parameters that, to our knowledge, have not been previously explored when it comes to long-term interventions trying to reduce sedentary behaviour; these include fMRI-response, cognitive function, or stress hormone levels. These measurements may provide further insights on the health effects of decreasing sedentariness.

Other strengths of the study are the randomization procedure and the long-term follow-up of 13 months. Time spent walking, standing, and sitting, as well as daily physical activity at different intensity levels, will be calculated during both the entire day and during working and non-working hours. The analysis will therefore show if the intervention with active workstations has any effects on sedentary time and activity levels not only at work, but also outside of working hours.

The study group consists of overweight or obese office workers, a group that is at an increased risk of developing different lifestyle-associated diseases linked to high amounts of sedentary time [19]. Levine et al. reported that obese individuals tend to spend 2.5 h more time per day sedentary compared to normal weight individuals [41]. Clinically, this is an important target group.

We need to find solutions that can target sedentary behaviour at work, and are feasible over long time periods. Active workstations could be one of these solutions.

## Abbreviations

BDNF: Brain-derived neurotrophic factor
BMI: Body mass index
CPGS: Chronic pain grade questionnaire
fMRI: Functional magnetic resonance imaging
HAD scale: Hospital anxiety and depression scale
HDL: High density lipoprotein, LDL, Low density lipoprotein
LPA: Light intensity physical activity
MVPA: Moderate- to vigorous physical activity
NEAT: Non-exercise activity thermogenesis
RCT: Randomized controlled trial
s-ED: self-rated exhaustion disorder
TMT: Trail making test
WSQ: Workforce sitting questionnaire
